# The role of emotional engagement and mood valence in retrieval fluency of mood incongruent autobiographical memory

**DOI:** 10.3389/fpsyg.2014.00083

**Published:** 2014-02-07

**Authors:** Jonathan Greenberg, Nachshon Meiran

**Affiliations:** Department of Psychology and Zlotowski Center for Neuroscience, Ben-Gurion University of the NegevBeer-Sheva, Israel

**Keywords:** emotional engagement, valence, emotion regulation, mood incongruent retrieval, fluency of autobiographical memories

## Abstract

**Background:** Retrieval of opposite mood autobiographical memories serves emotion regulation, yet the factors influencing this ability are poorly understood.

**Methods**: Three studies examined the effect of mood valence (sad vs. happy) and degree of emotional engagement on fluency of mood incongruent retrieval by manipulating emotional engagement and examining the effect of emotional film clips on the Fluency of Autobiographical Memory task.

**Results**: Following both sad and happy film clips, participants who received emotionally engaging instructions exhibited a greater recall latency of the first opposite mood memory, and had generated less such memories than those receiving emotionally disengaging instructions (Studies 1 and 2). A happy mood induction resulted in recollection of fewer mood incongruent memories compared to a sad mood induction. Providing emotionally engaging instructions was found to specifically hinder mood incongruent retrieval, without impairing mood congruent retrieval (Study 3).

**Conclusion**: High emotional engagement seems to impair the retrieval of mood incongruent memories. Being in a happy mood may also partially impair such retrieval. Implications regarding emotional regulation are discussed.

## Introduction

Imagine your negative mood after failing an exam or after receiving a rejection letter from a journal. One way to boost your mood after such an event might be to recall the high grade you have recently received, an article that has been accepted recently to a prestigious journal, or the warm hug you received from someone you love. It is your ability to recall these mood incongruent memories that may dictate the success in down-regulating the negative mood.

The ability to recall mood related memories has been studied as a part of the more general issue concerning the relation between mood and memory. A common way of exploring this relationship has been to compare the retrieval of memories that match the current mood (mood congruent recall) with retrieval of memories opposite in valence to the current mood (mood incongruent recall; for reviews see Blaney, 1986; Singer and Salovey, 1988; Matt et al., 1992; Rusting, 1999; Barry et al., 2004). In this work, we are interested in the latter, and focus primarily on the ability to retrieve mood incongruent autobiographical memories.

As the example above suggests, retrieving mood incongruent autobiographical memories seems of particular importance first and foremost due to its role in the regulation of emotion (see Isen et al., 1978; Isen, 1984; Rusting and DeHart, 2000). In a series of experiments performed by Parrott and Sabini (1990), individuals in a sad mood recalled happier memories than individuals in a happy mood. This was evident both in field quasi-experiments (e.g., comparing memory valence of students who received a bad grade vs. a good grade and comparing memory valence of individuals on cloudy days vs. sunny days) and in three lab experiments involving mood induction. Similarly, in a study by Josephson (1996), following either sad or neutral mood inductions participants were requested to recall two autobiographical memories, and choose them to be either positive or negative in valence. The majority of participants in the sad condition recalled a happy second memory, and most of them explicitly stated that they deliberately recruited those happy memories in order to counteract their negative moods. Thus, mood incongruent retrieval seems an important and common means of emotion regulation. A second reason for the importance of focusing on mood incongruent retrieval regards its relation to depression (e.g., Blaney, 1986). Previous findings have indicated that depressed individuals tend to use maladaptive emotion regulation strategies (Campbell-Sills et al., 2006; Garnefski and Kraaij, 2006), yet a recent study has found that despite having a difficulty in benefitting from mood incongruent recall when focusing of the memories in an abstract and ruminative manner, depressed individuals significantly improved their moods by retrieving and focusing on mood incongruent memories in a concrete and non-analytical manner (Werner-Seidler and Moulds, 2012).

The question addressed in this work is what dictates the retrieval fluency of opposite mood memories? While previous research has suggested several correlates of emotional memory retrieval in general, such as personality traits of neuroticism and extraversion (Denkova et al., 2012) and increased brain activity in regions such as the amygdala, hippocampus, and several regions in the prefrontal cortex (see Svoboda et al., 2006; Denkova et al., 2013; Young et al., 2013), the factors that specifically influence the retrieval of memories that are opposite to the current mood are still relatively poorly understood. Some authors (Isen, 1985; Singer and Salovey, 1988; Rusting, 1998) have suggested that mood incongruent memories are less accessible when in a happy mood than in a sad mood. Others have suggested that such difficulty in retrieval of mood incongruent happy autobiographical memories actually reflects levels of sadness (Sheppes and Meiran, 2007). In the current work we examined these two valence-specific hypotheses, along with an alternative hypothesis suggesting that *emotional engagement* plays a central role in determining how mood influences the fluency of retrieval of mood incongruent autobiographical memories. We refer to emotional engagement here as the extent to which one is involved in the emotional experience. We note that although the degree of emotional engagement may be related to emotional intensity, the two are not synonymous, as one may feel intense emotion, yet may turn away and disengage from it [e.g., through application of emotion regulation strategies such as suppression or response modulation (see Gross, 2001)].

The plausibility of emotional engagement as a determining factor in the retrieval of mood incongruent memories may be illustrated by drawing an analogy between mood incongruent retrieval and task switching (see Greenberg and Meiran, 2013). The task switching paradigm (see Kiesel et al., 2010; Meiran, 2010; Vandierendonck et al., 2010 for reviews) involves asking participants to rapidly switch between different tasks including speeded choice tasks, memory retrieval tasks, and so forth. There is a relatively wide agreement among researchers that this switching involves a behavioral cost, due to the need to change the task-set, which may be described as the configuration of the cognitive system needed in order to perform the task. Moreover, evidence suggests that these costs are caused (perhaps in part) by the difficulty in disengaging from the previously relevant task set. A particular finding which supports this conclusion is “switch asymmetry,” which refers to the fact that the more engaging a task is (or appears to be, see Barutchu et al., 2013), the longer it takes to disengage from it in order to switch to a different task (e.g., Allport et al., 1994; Yeung and Monsell, 2003). Note that mood resembles task sets in many respects. Like task sets, mood prepares the organism to act and think in a manner appropriate for the given context. The analogy between task sets and mood suggests that the greater the engagement in a particular mood, the “deeper” one is immersed in it and allocates attention resources to it, and the more difficult it would be to switch to a different mood. As a result, greater mood engagement is expected to impair the subsequent retrieval of opposite mood memories (see Bower, 1981).

In Study 1, we examined the effect of emotional engagement on retrieval fluency of mood incongruent memories by manipulating emotional engagement in two different ways: providing instructions to engage vs. disengage emotionally and manipulating the duration of the clips used (long vs. short) to elicit the emotion. Our dependent measure was based on participants' performance on the Fluency of Autobiographical Memory (FAM) task (Sheppes and Meiran, 2007; Greenberg and Meiran, 2013) in which participants are asked to recall as many autobiographical events as possible that are linked to the opposite-to-induced emotion. Sheppes and Meiran examined two dependent measures: recall latency of the first opposite mood memory, and the total number of memories recalled. In their study, the generation of positive autobiographical memories was found to be highly sensitive to emotion manipulation (negatively valenced vs. positively valenced film clips), with partial μ^2^ values exceeding 0.30.

Sheppes and Meiran (2007), who examined recollection of happy memories, claimed that FAM indexes the degree of sadness. If this were the case, less fluency (a greater latency in recalling the first opposite mood memory and a smaller number of total generated memories) is expected following a sad film compared to a happy film. Since the sad valence of the mood in considered to interfere with mood incongruent retrieval as measured by the FAM, we consider this prediction in a “sad valence hypothesis.” Conversely, if retrieval of mood incongruent memories is more difficult following happy moods (Isen, 1985; Singer and Salovey, 1988; Rusting, 1998) a reversed pattern is to be expected, with less fluency following a happy film, a prediction which we consider in a “happy valence hypothesis.” Additionally, if degree of emotional engagement is a significant determining factor in mood incongruent retrieval, an alternative outcome is predicted via an “engagement hypothesis,” in which less fluency is expected following high levels of emotional engagement, regardless of mood valence (negative or positive). Following the analogy to the task switching paradigm, we consider the latency in first opposite mood memory generation as the primary index for emotional engagement, as this latency assesses the time needed initially disengage from the current mood. The total number of opposite mood memories generated indexes fluency of memories *after* the initial disengagement, which has previously been suggested as an index for “recovery speed” from the emotional experience (see Greenberg and Meiran, 2013).

Study 1 examined the effects of emotional engagement on mood incongruent retrieval following sad and happy mood inductions in a set order (sad induction first, happy induction second). Study 2 used a similar procedure as Study 1 using only a happy mood induction, to control for potential order effects. Study 3 examined the effect of mood inductions on mood congruent rather than incongruent retrieval to assess whether effects of emotional engagement on autobiographical memory retrieval are specific to incongruent memories.

## Study 1

### Method

#### Participants

Participants were 42 undergraduate students studying at Ben-Gurion University (35 women, mean age 23.74, *SD* = 2.92). Participants were recruited via posters around the university campus, and were assigned to the four groups according to the order in which they entered the experiment. The four groups were formed by the factorial combination of 2 independent variables—Instructions Type (engaging vs. disengaging) and Film Clip Duration (long vs. short). Data of one male participant were excluded due to a technical error. Data from one female participant were excluded from the FAM recall latency analysis due to her failure to press the space bar upon recollection of the first memory, which prevented her RT logging. The FAM RT data that were eventually analyzed were from 10 participants in each group. The four groups did not differ in age [*F*_(3, 36)_ = 1.01, *ns*] or gender (minimal *p* = 0.46, Fisher's exact test).

#### Film stimuli

Films were taken from Sheppes and Meiran (2007). The sadness eliciting film was a documentary depicting the story of a soldier killed in military action and his bereaved relatives and girlfriend. The film was edited to create a long (2:21 min) and a short (0:33 min) version for the two clip length conditions. The happiness/amusement eliciting film lasted 5:19 min and consisted of 2 scenes from a well known comedy duo act in a program called “The Assi and Guri show,” in which the two comedians pantomime a live music band and later on perform an “interview” in which one elaborates on his “fake” and eccentric alien abduction experiences. This film was edited as well to create a long (5:19 min) and a short (0:39 min) version. The short versions were edited like trailers and summarized the long versions while including most of the information. The happy film has targeted primarily humor and amusement, yet the film has been validated to illicit self reported happiness. Likewise, the sad film has been validated to illicit self reported sadness (Sheppes and Meiran, 2007). The happy and sad clips were unequal in length. This, however, does not pose a problem for our study since according to the positive and negative valence hypotheses, the two kinds of films are supposed to yield opposing effects on autobiographical memory retrieval fluency while according to the engagement hypothesis, the influence on memory retrieval should not depend on valence. We will return to this point in the General Discussion.

#### Measures

***Self reported emotion ratings.*** At the beginning of the experiment as well as immediately following each film clip, participants were asked to rate their general emotional mood (from “very bad mood” to “very good mood”) as well as degree of happiness and sadness on a scale ranging from 1 to 9, as a measure of emotion intensity.

***FAM.*** FAM instructions were similar to those used by Sheppes and Meiran (2007), with the addition of recollection of sad memories following the happy film (see Greenberg and Meiran, 2013). Following the film clips, participants completed the self reported mood ratings. They were then asked to recall a specific personal happy memory (or a specific sad one following the happy film clip). The specific valence (sad or happy) of the memory to be recalled was mentioned at the end of the FAM instructions in order to prevent participants from starting to recall memories prior to the start of the time clock. Upon recollection participants pressed the space bar (in order for the computer to log the time to recall the memory), wrote down a key word summing up the event (which they would later use to describe the memory in detail) and continued to recall the next memory until 2:40 min had elapsed. The 2:40 min limit was determined by Sheppes and Meiran based on pilot data indicating that very few additional memories were retrieved after this time. The participants were then asked to provide a relatively detailed description of each of the memories next to each of the keywords they had written, in order to ensure that the memories fit the required mood and that they were different from one another. This was explained to the participants in advance.

#### Emotional engagement instructions

Prior to each emotion induction, participants in all four groups received oral instructions which they were asked to execute while watching the upcoming film clip. The groups receiving engaging instructions were given the following instructions in Hebrew (the participants' native language): “Please attend carefully to the following film clip. If any emotions or feelings arise while watching, please allow yourself to fully experience these feelings, without distancing or blocking yourself from them in any way.” The groups receiving disengaging instructions were given the following instructions: “Please attend carefully to the following film clip, but watch it in a non-emotional manner. Try to view the film objectively and neutrally, paying attention to the facts but keeping yourself emotionally distant and uninvolved.” These instructions were given twice to each participant, once before each mood inducing film.

#### Procedure

After signing an informed consent form, participants completed the following tasks: They completed a baseline self reported mood check of sadness, happiness, and general mood, then received emotional engagement/disengagement instructions, watched the sad mood film (short or long depending on the condition assigned to the group), completed another self reported mood check, performed the FAM task (of happy memories), received the same viewing instructions as before, watched a happy film (short or long depending on the condition assigned to the group), completed a final self reported mood check followed by a another FAM task (of sad memories), and were then debriefed. We did not counterbalance film clips primarily to standardize testing order in an effort to maximize statistical power.

### Results and discussion

The results were analyzed using Alpha = 0.05. In examining effects with a predicted direction (according to each of the three main hypotheses detailed in the Introduction section) we employed confirmatory one-tailed *t*-test contrasts by using the Analysis of variance (ANOVA) MSes (converting the ANOVA scores into *t*-scores enabled the one-tailed examination of the hypotheses with a predicted direction). Other (non-hypothesized) effects are reported using standard exploratory ANOVA analyses (as in all experiments hereby reported).

#### Time to generate the first memory

**Figure 1:**
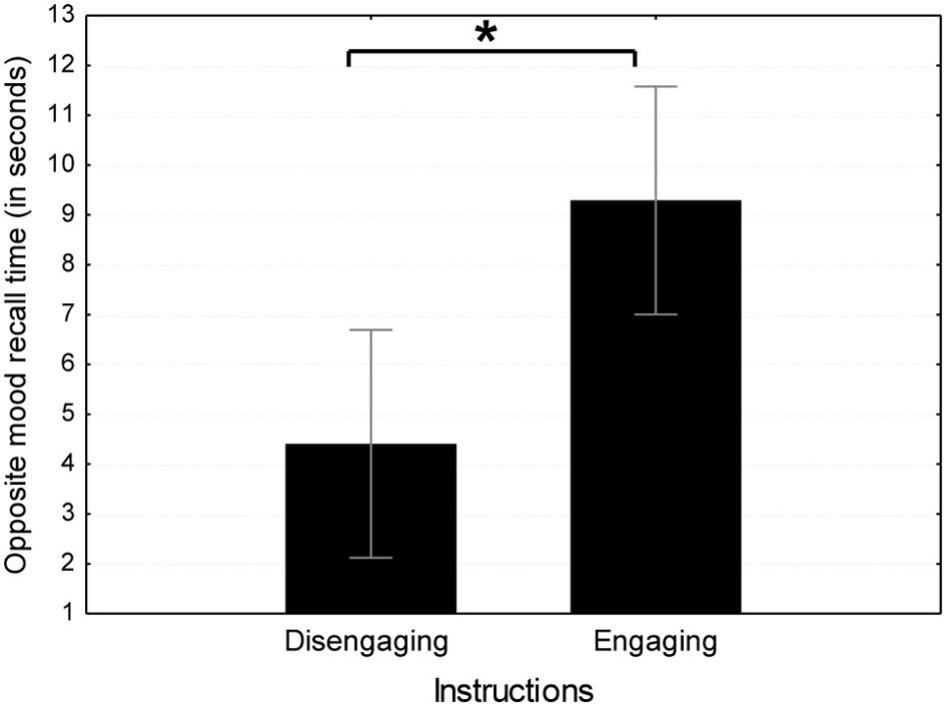
**Mean time to generate the first opposite mood memory in emotionally engaging instruction conditions in Study 1.** ^*^p < 0.05.

A mixed model ANOVA was conducted on the time to generate the first memory, with the between-subjects independent variables being Instructions Type (engaging vs. disengaging) and Clip Length (long vs. short) and the within subjects variable Mood Induction Type (happy vs. sad). Using the appropriate MSes from the ANOVA, we examined the one-tailed contrast *t*-tests according to each hypothesis. According to the “engagement hypothesis,” the time to generate the first opposite memory (the primary measure for emotional engagement as detailed in the Introduction section) should be longer for participants in the emotionally engaging conditions compared to participants in emotionally disengaging conditions. Results from emotionally engaging instructions support this hypothesis, as participants in the emotionally engaging instructions condition (*M* = 9.29 s, *SD* = 3.24) took more than twice the time to generate the first opposite mood memory than those in the emotionally engaging instructions condition [*M* = 4.4 s, *SD* = 8.77; *t*_(36)_ = 3.06, *p* < 0.01; see Figure 1]. This effect of emotional engagement via instructions was not qualified by an interaction with Induction Type [*F*_(1, 36)_ = 0.13, *ns*] and was statistically comparable for happy memories following the sad induction [*t*_(36)_ = 2.18, *p* < 0.05] and sad memories following the happy induction [*t*_(36)_ = 2.41, *p* = 0.01]. No differences were found between clip length conditions [*t*_(36)_ = 0.95, *ns*].

According to the “happy valence hypothesis,” the time to generate the first opposite memory should be significantly longer following the happy film compared to the sad film. According to the “sad valence hypothesis” the opposite is predicted. In contrast to both of these hypotheses, results indicate that the time to generate the first opposite memory following the happy film (*M* = 7.06 s, *SD* = 7.03) did not significantly differ than that of the sad film [*M* = 6.64 s, *SD* = 7.06; *t*_(36)_ = 0.30, *ns*]. No other main effects or interactions in the exploratory ANOVA approached significance (Maximal *F* = 0.90, *ns*). Thus, opposite mood memory generation time results support the contribution of emotional engagement but do not support the contribution of specific valence in mood incongruent retrieval.

#### Total number of memories generated

**Figure 2:**
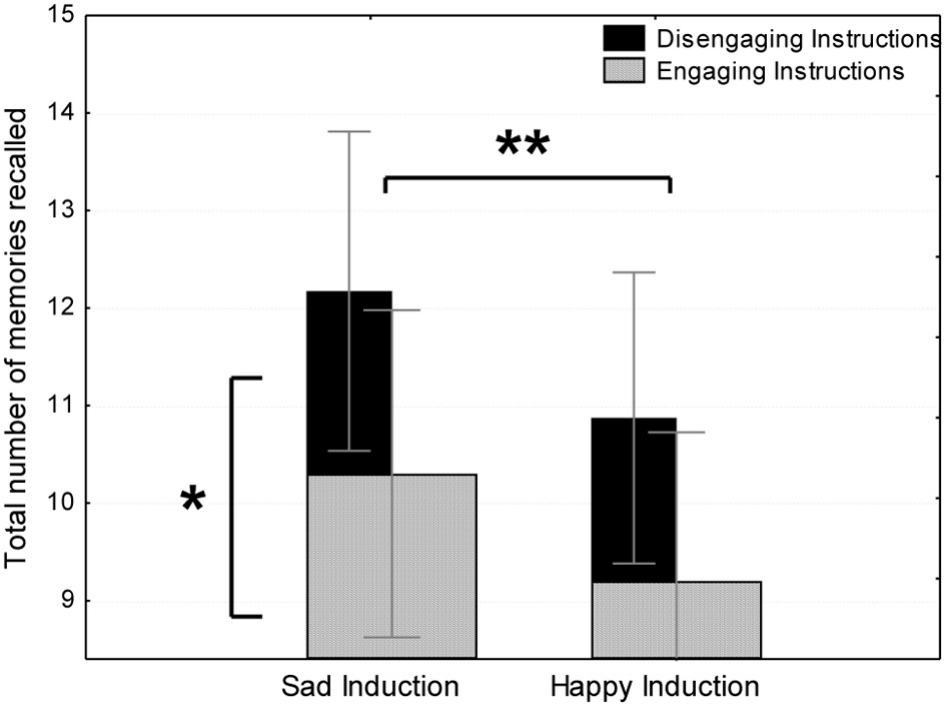
**Total number of memories recalled in emotionally engaging instruction and mood induction conditions in Study 1 (The figure shows that the increase in the number of recalled memories due to disengaging instructions was roughly the same for sad and happy mood induction).** ^*^A significant main effect for instruction type: more opposite mood memories recalled following emotionally disengaging instructions than following emotionally engaging instructions. ^**^A significant main effect for mood induction: more opposite mood memories recalled following a sad induction than following a happy induction.

We performed a Three-Way mixed ANOVA with the independent variables Instruction Type (engaging vs. disengaging) and Clip Length (long vs. short) as between subjects variables and Mood Induction Type (happy vs. sad) as the within subject variable. The total number of memories indexes the fluency of memories after the initial disengagement from the emotion, and thus, is a secondary rather than primary measure of emotional engagement. Nevertheless, according to the “engagement hypothesis” participants in emotionally engaging conditions are expected to recall fewer memories than participants in the emotionally disengaging conditions. Once again, results from emotionally engaging instructions support this hypothesis, as participants in the emotionally engaging instructions condition (*M* = 9.75 memories, *SD* = 2.92) recalled fewer memories than those in the emotionally disengaging instructions condition [*M* = 11.52, *SD* = 3.60; *t*_(37)_ = 1.69, *p* < 0.05; see Figure 2]. This effect was prevalent regardless of Induction Type, as indexed by the non-significant interaction [*F*_(1, 37)_ = 0.08, *ns*]. Nonetheless, the effect of Instruction Type within each emotion failed reaching statistical significance, probably due to lack of statistical power [*t*_(37)_ = 1.62, 1.59, *p* = 0.06 after the sad clip and happy clip, respectively]. No differences were found between clip length conditions [*t*_(37)_ = 0.50, *ns*].

According to the “happy valence hypothesis,” fewer memories are expected to be recalled following the happy film compared to the sad film. According to the “sad valence hypothesis” the opposite is predicted. Results indicate that fewer mood incongruent memories were recalled after the happy film (*M* = 10.05, *SD* = 3.37) than after the happy film [*M* = 11.24, *SD* = 3.71; *t*_(37)_ = 3.37, *p* < 0.01; see Figure 2], regardless of Instruction Type or Clip Length. No other effect or interaction approached significance (Maximal *F* = 1.97, *ns*).

Thus, results from total number of memories recalled provide additional and converging support for the “engagement hypothesis” using emotionally engaging instructions, and additionally provide some evidence supporting the “happy valence hypothesis.”

#### Self reported mood checks

Means are listed in Table 1. No significant differences were found between Instruction Type conditions or between Clip Length conditions in baseline pre-induction measures of happiness, general mood, or sadness (maximal *t* = 0.57, *ns*). A Multivariate ANOVA^1^ (MANOVA) of Instruction Type (engaging vs. non-engaging), Clip Length (long vs. short), and Induction Type (pre-induction, sad induction, happy induction) as the independent variables, and ratings on the three Rating Types (happy, general mood, sad) as the dependent variables was conducted.

**Table 1:** **Means and *SD*'s (in parenthesis) of self reported happiness, general mood, and sadness ratings in Study 1, in the different emotional engagement conditions**.

		**Baseline**	**Post sad induction**	**Post happy induction**
Engaging instructions	Happiness	6.25 (0.91)	3.95 (1.70)	6.95 (1.32)
	Gen. mood	6.80 (1.00)	4.75 (1.45)	7.30 (0.92)
	Sadness	2.65 (1.93)	5.55 (1.93)	2.10 (1.29)
Disengaging instructions	Happiness	6.14 (1.42)	5.10 (1.64)	6.62 (1.36)
	Gen. mood	6.57 (1.47)	5.81 (1.40)	6.90 (1.49)
	Sadness	2.90 (2.05)	3.71 (2.08)	2.38 (1.75)
Long clip	Happiness	6.24 (0.94)	3.95 (1.86)	6.76 (1.48)
	Gen. mood	6.76 (0.83)	4.71 (1.45)	7.29 (1.06)
	Sadness	2.62 (1.91)	5.25 (2.34)	2.00 (1.22)
Short clip	Happiness	6.15 (1.42)	5.15 (1.42)	6.80 (1.20)
	Gen. mood	6.60 (1.60)	5.90 (1.33)	6.90 (1.41)
	Sadness	2.95 (1.06)	4.00 (1.89)	2.50 (1.79)

Scales of all measures range from 1 to 10.

To check our manipulation, the main effect for Induction Type was examined and found to be significant [Wilks' Lambda = 0.21, *F*_(6, 32)_ = 19.71, *p* < 0.001]. Subsequent univariate ANOVAs revealed that both happy [*F*_(1, 37)_ = 73.94, *p* < 0.001] and general mood ratings [*F*_(1, 37)_ = 43.44, *p* < 0.001] were lower, and sad ratings [*F*_(1, 37)_ = 28.60, *p* < 0.001] were higher, after a sad mood induction than at baseline (pre-induction) level. The opposite was found when comparing ratings following a happy induction to ratings following a sad induction, with happy [*F*_(1, 37)_ = 123.84, *p* < 0.001] and general mood ratings [*F*_(1, 38)_ = 65.50, *p* < 0.001] being higher, and sad ratings [*F*_(1, 37)_ = 74.56, *p* < 0.001] being lower after a happy induction. These indicate that mood inductions elicited the desired moods.

The MANOVA also yielded a significant interaction of Induction Type (pre-induction, sad induction, happy induction) × Instruction Type (engaging vs. non-engaging) [Wilks' Lambda = 0.66, *F*_(6, 32)_ = 2.80, *p* < 0.05]. The following univariate ANOVAs indicated that self reported moods of participants who received emotionally engaging instructions were significantly more affected than those who received disengaging instructions, on all rating types, in all induction types. Specifically, following a sad mood induction, participants who received emotionally engaging instructions exhibited a greater decline in happy [*F*_(1, 37)_ = 10.71, *p* < 0.01] and general mood [*F*_(1, 37)_ = 9.85, *p* < 0.01] ratings, and a greater incline in sad rating [*F*_(1, 37)_ = 9.40, *p* < 0.01] than participants who received emotionally disengaging instructions, compared to baseline mood ratings. In contrast, following a happy mood induction, participants who received emotionally engaging instructions exhibited a greater incline in happy [*F*_(1, 37)_ = 13.43, *p* < 0.001] and general mood [*F*_(1, 37)_ = 11.08, *p* < 0.01] ratings, and a greater decline in sad rating [*F*_(1, 37)_ = 14.90, *p* < 0.001] than participants who received emotionally disengaging instructions, compared to ratings following the sad induction.

The third and final significant effect found in the MANOVA was that of Induction Type × Clip Length (short vs. long) [Wilks' Lambda = 0.66, *F*_(6, 32)_ = 2.69, *p* < 0.05]. Subsequent ANOVAs indicated that following a sad induction, participants who viewed a long clip exhibited a greater decline in happy [*F*_(1, 37)_ = 11.81, *p* < 0.01] and general mood [*F*_(1, 37)_ = 10.68, *p* < 0.01], and a greater incline in sad ratings [*F*_(1, 37)_ = 5.30, *p* < 0.05], than participants who viewed a short clip, compared to baseline ratings. In contrast, following a happy induction, participants who viewed a long clip exhibited a greater incline in happy [*F*_(1, 37)_ = 8.95, *p* < 0.01] and general mood [*F*_(1, 37)_ = 13.06, *p* < 0.001] ratings, and a greater decline in sad ratings [*F*_(1, 37)_ = 10.25, *p* < 0.01] than participants who viewed a short clip, compared to ratings following the happy mood induction.

Thus, since participants who received emotionally engaging instructions had exhibited more than double the recall latency of the first opposite mood memory, and generated overall less opposite mood memories than participants who received emotionally disengaging instructions, results of the current study support the emotional engagement hypothesis, and suggest that high levels of emotional engagement hinder mood incongruent retrieval, compared to low levels of emotional engagement. Weaker support was found for the “happy valence hypothesis,” as participants overall recalled fewer opposite mood memories following a happy film compared to a sad film, yet this hypothesis was not supported using the first memory recall latency measure. No support was found for the “sad mood hypothesis.”

Manipulating emotional engagement through length of emotion eliciting film clips did not significantly influence mood incongruent retrieval. One possible *post-hoc* explanation for this is that in our study, in an attempt to maintain clip coherence, short clips became “condensed” versions of long clips thereby maintaining high emotional “potency,” which has been previously shown to inhibit mood incongruent recall (Rinck et al., 1992). Another possible explanation is that a floor effect occurred since in order to insure a significant emotional effect, both the durations we had used may be considered rather “long” (minimum of 0:33 min) compared to other studies eliciting emotion (e.g., Isen et al., 1978; Sakaki, 2007; Ball and Hennessey, 2009).

## Study 2

Results of Study 1 demonstrated that emotional engagement hindered mood incongruent autobiographical retrieval following both sad and happy film clips. Film order in Study 1was fixed (sad film first, happy film second) in an effort to maximize statistical power. It thus, may be claimed that performance on the FAM after the happy film (which came second) might have been affected by viewing the first (sad) film or by the recent recollection of happy memories. The aim of Study 2 was to provide a firmer base for the results obtained in Study 1 regarding the effects of emotional engagement on mood incongruent retrieval (i.e., the “engagement hypothesis”) by ruling out the possibility of such order effects via administering the FAM for mood incongruent memories following a happy film at the beginning of the experiment.

### Method

#### Participants

Participants were 37 undergraduate students studying at Ben-Gurion University (24 women, mean age 24.05, *SD* = 1.61). Since Clip Length did not affect FAM performance in Study 1, participants in this study were assigned to only two Instruction Type groups (emotionally engaging instruction group vs. an emotionally disengaging instruction group), according to the order in which they entered the experiment. Data from three participants were excluded from FAM analysis due to their failure to press the space bar upon recollection of the first memory, which prevented her RT logging. FAM data were analyzed were from 18 participants from the emotionally engaged group, and 16 participants from the disengaged group. The two groups did not differ in age [*t*_(32)_ = 1.29, *ns*] nor in gender (*p* = 0.08, Fisher's exact test).

#### Film stimuli

The only film used was the longer version (5:19 min) of the happy film used in Study 1, from the Israeli standup comedy act “The Assi and Guri show.”

#### Measures

Self reported emotion ratings, FAM, and emotionally engaging and disengaging instructions were all identical to those in Study 1, yet were used only for a happy film.

#### Procedure

The procedure was identical to that of Study 1, apart for the fact that the only film used was the happy film and the only FAM administered was that of sad memories.

### Results and discussion

#### Time to generate the first memory

**Figure 3:**
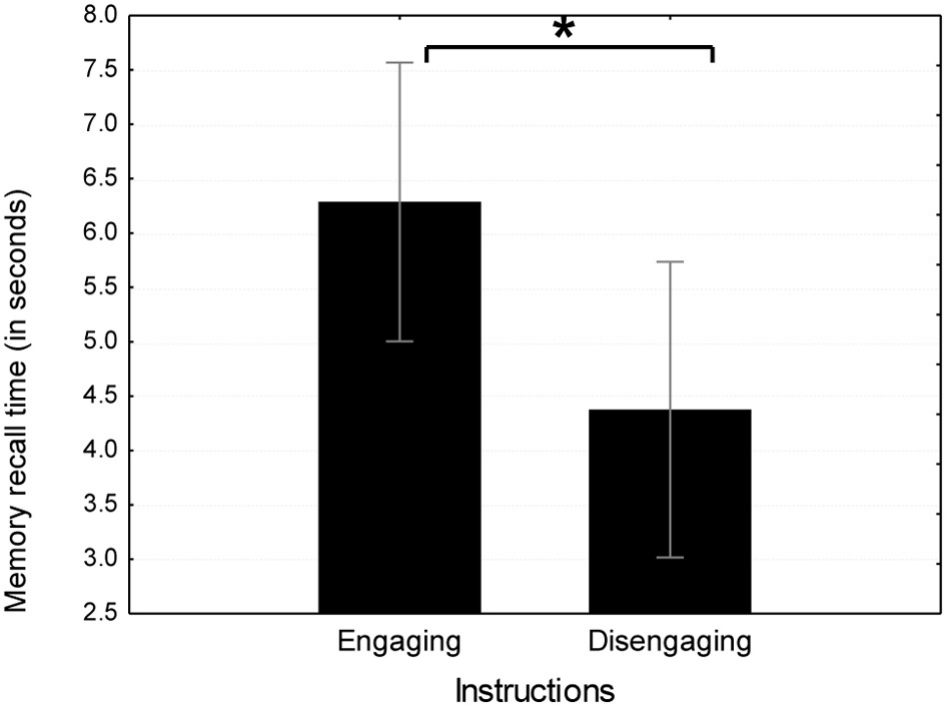
**Mean time to generate the first opposite mood memory in emotionally engaging instruction conditions in Study 2.** ^*^p < 0.05.

A *t*-test was conducted comparing the two instruction type groups in the time to generate the first opposite mood memory. As predicted by the “engagement hypothesis,” the emotionally engaging instruction group (*M* = 6.29 s, *SD* = 3.43) took significantly longer to generate the first sad memory than the emotionally disengaging instruction group [*M* = 4.37 s, *SD* = 1.36; *t*_(32)_ = 2.08, *p* < 0.05; see Figure 3].

#### Total number of memories generated

**Figure 4:**
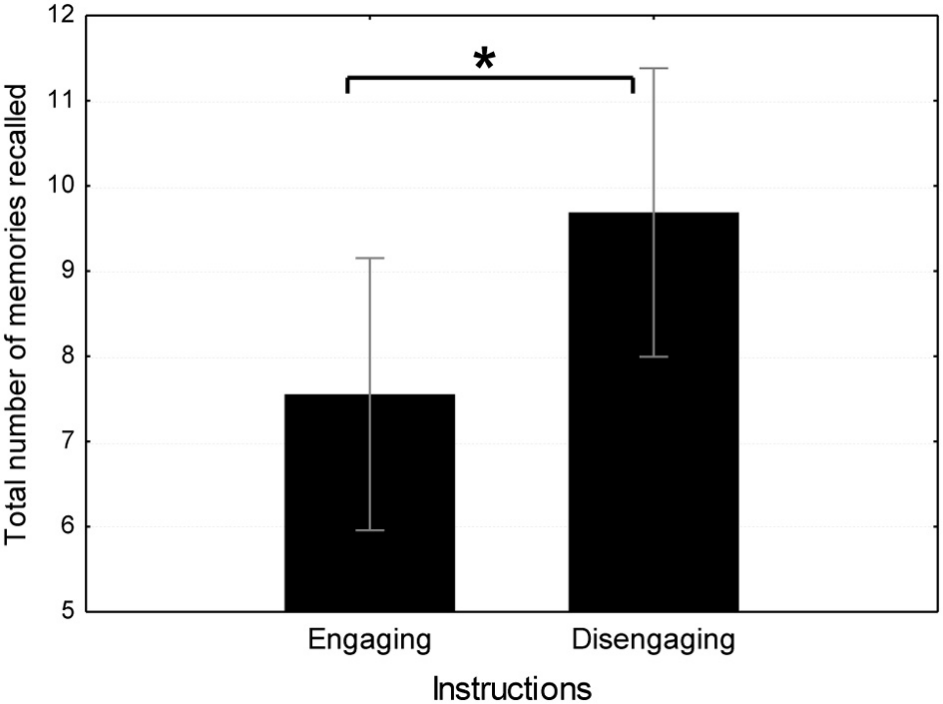
**Total number of memories recalled in emotionally engaging instruction conditions in Study 2.** ^*^p < 0.05

In a similar *t*-test, the emotionally engaging instruction group (*M* = 7.56, *SD* = 2.45) generated less sad memories following the happy film than the emotionally disengaging instruction group [*M* = 9.69, *SD* = 4.09; *t*_(32)_ = 1.87, *p* < 0.05; see Figure 4].

The fact that the current study obtained similar results to those obtained in Study 1 regarding emotional engagement following a happy mood induction, yet without previous experience with a sad mood induction or with retrieval of happy memories rule out order effects as an alternative account for the results of Study 1.

#### Self reported mood checks

Means are listed in Table 2. No significant differences were found between groups in baseline pre-induction measures of happiness, general mood, or sadness (maximal *t* = 1.23, *ns*). A MANOVA of Instruction Type group (engaging instructions vs. disengaging instructions) and Induction (pre-induction, happy induction) as the independent variables, and ratings on the three Rating Types (happy, general mood, sad) as the dependent variables was conducted. A main effect was found for Induction [Wilks' Lambda = 0.59, *F*_(3, 33)_ = 7.58, *p* = 0.001]. Subsequent univariate ANOVAs revealed that both happy [*F*_(1, 35)_ = 16.04, *p* < 0.001] and general mood ratings [*F*_(1, 35)_ = 21.27, *p* < 0.001] were higher, and sad ratings [*F*_(1, 35)_ = 7.35, *p* = 0.01] were lower, after the happy mood induction than at baseline (pre-induction) level. This indicates that the mood induction elicited the desired moods.

**Table 2:** **Means and *SD*'s (in parenthesis) of self reported happiness, general mood, and sadness ratings in Study 2, in the different emotional engagement conditions**.

		**Baseline**	**Post happy induction**
Engaging instructions	Happiness	6.11 (2.05)	7.17 (1.54)
	Gen. mood	6.11 (2.00)	7.61 (1.61)
	Sadness	2.83 (2.15)	2.22 (1.77)
Disengaging instructions	Happiness	6.11 (1.24)	6.79 (1.55)
	Gen. mood	6.63 (1.38)	7.11 (1.29)
	Sadness	2.21 (0.92)	1.79 (1.23)

Scales of all measures range from 1 to 10.

The mood induction did not affect self reported mood checks of the two groups differently. This has been evident by the non-significant interaction between Induction (pre-induction vs. happy induction) and group (engaging instructions vs. disengaging instructions) [*F*_(3, 33)_ = 2.13, *ns*].

Self reported mood checks require that participants rate the intensity of each emotion on a scale ranging from 1 to 9. The fact that the groups differed in FAM performance but not differ in self-reported mood checks provides evidence that emotional engagement and emotion intensity are separate and independent from one another. Although high emotional engagement *may* be accompanied by high levels of emotional intensity (see Study 1 and 3), this is not always the case, as evident in the current study. The fact that the groups differed in FAM performance but not in self-reported mood checks supports the notion that impairments in mood incongruent retrieval were not due to the intensity of the emotion but rather due to emotional engagement.

## Study 3

Results of Studies 1 and 2 demonstrated that high levels of emotional engagement hinder mood incongruent autobiographical retrieval. The aim of Study 3 was to validate that this hindrance is specific to mood incongruent autobiographical retrieval rather than a hindrance to autobiographical retrieval in general. To this end, participants received emotionally engaging or disengaging instructions and viewed emotion eliciting films as in Study 1, yet the FAM measure was used to examine mood *congruent* rather than incongruent memories. The claim that high levels of emotional engagement hinder autobiographical memory retrieval in general (rather than only mood incongruent retrieval) leads to the hypothesis that that, as in Study 1and 2, participants receiving emotionally engaging instructions would take longer to generate the first mood congruent memory, and/or will generate less such memories than those receiving disengaging instructions.

### Method

#### Participants

Participants were 28 undergraduate students studying at Ben-Gurion University (14 women, mean age 25.14, *SD* = 1.21). As in Study 2, participants assigned to only two groups (emotionally engaging instruction group vs. emotionally disengaging instruction group) of 14 participants each, according to the order in which they entered the experiment. The two groups did not differ in age [*t*_(26)_ = 1.26, *ns*] nor in gender (*p* = 0.65, Fisher's exact test).

#### Film stimuli

The films used were the longer versions of the sad (story of a soldier killed in military action) and happy (the Israeli standup comedy act “The Assi and Guri show”) films used in Study 1.

#### Measures

Self reported emotion ratings, FAM, and emotionally engaging and disengage instructions were all identical to those in Study 1, with the exception of retrieving mood congruent rather than incongruent memories (i.e., recalling sad memories following the sad film and happy memories following the happy film).

#### Procedure

The procedure was identical to that of Study 1, as was film order (sad film first, happy film second).

### Results and discussion

#### Time to generate the first memory

A Two Way ANOVA was conducted with Instruction Type (engaging vs. disengaging) and Mood Induction Type (sad vs. happy) as the independent variables and time to generate the first memory as the dependent variable. According to the hypothesis that high emotional engagement hinders autobiographical memory retrieval in general, participants receiving emotionally engaging instructions should take longer to generate the first mood congruent memory. This hypothesis was not supported, as no differences were found between emotional engagement conditions [*t*_(26)_ = 0.96, *ns*]. Moreover, an opposite trend to that of Studies 1 and 2 was found, in which participants receiving emotionally engaging instructions (*M* = 4.24 s, *SD* = 1.64) were (non-significantly) *quicker* than those receiving emotionally disengaging instructions (*M* = 5.73 s, *SD* = 5.58) in retrieving the first memory. No other effects approached significance in the ANOVA [Maximal *F*_(1, 26)_ = 0.92, *ns*].

#### Total number of memories generated

A similar ANOVA was conducted with Total Number of Memories as the dependent variable. Once again, no support was found for the hypothesis that high emotional engagement hinders autobiographical memory retrieval in general, as no differences were found between emotional engagement conditions [*t*_(26)_ = 1.00, *ns*]. Moreover, an opposite trend to that of Studies 1 and 2 emerged, in which those receiving emotionally engaging instructions (*M* = 9.43, *SD* = 2.62) generated (non-significantly) *more* memories than those receiving emotionally disengaging instructions [*M* = 8.29, *SD* = 3.40].

A main effect was found for Mood Induction Type [*F*_(1, 26)_ = 5.37, *p* < 0.05], in which more happy (*M* = 9.43, *SD* = 3.53) than sad (*M* = 8.29, *SD* = 3.08) memories were generated, regardless of Instruction Type.

#### Self reported mood checks

Means are listed in Table 3. No significant differences were found between Instruction Type conditions in baseline pre-induction measures of happiness, general mood, or sadness (maximal *t* = 1.31, *ns*). A Multivariate ANOVA (MANOVA) of Instruction Type (engaging vs. non-engaging), and Induction Type (pre-induction, sad induction, happy induction) as the independent variables, and ratings on the three Rating Types (happy, general mood, sad) as the dependent variables was conducted.

**Table 3:** **Means and *SD*'s (in parenthesis) of self reported happiness, general mood, and sadness ratings in Study 3, in the different emotional engagement conditions**.

		**Baseline**	**Post sad induction**	**Post happy induction**
Engaging instructions	Happiness	5.57 (1.16)	3.07 (1.54)	7.29 (1.44)
	Gen. mood	6.43 (1.50)	3.21 (1.19)	7.5 (1.29)
	Sadness	3.07 (1.64)	6.86 (1.51)	2.86 (1.70)
Disengaging instructions	Happiness	6.07 (1.43)	4.29 (1.90)	6.79 (1.25)
	Gen. mood	6.29 (1.54)	4.36 (1.74)	7 (1.24)
	Sadness	2.29 (1.54)	4 (2.15)	2.79 (1.72)

Scales of all measures range from 1 to 10.

A significant main effect was found for Induction Type [Wilks' Lambda = 0.167, *F*_(6, 21)_ = 17.47, *p* < 0.001]. Subsequent univariate ANOVAs revealed that both happy [*F*_(1, 26)_ = 46.52, *p* < 0.001] and general mood ratings [*F*_(1, 26)_ = 59.22, *p* < 0.001] were lower, and sad ratings [*F*_(1, 26)_ = 43.97, *p* < 0.001] were higher, after a sad mood induction than at baseline (pre-induction) level. The opposite was found when comparing ratings following a happy induction to ratings following a sad induction, with happy [*F*_(1, 26)_ = 80.56, *p* < 0.001] and general mood ratings [*F*_(1, 26)_ = 94.89, *p* < 0.001] being higher, and sad ratings [*F*_(1, 26)_ = 35.77, *p* < 0.001] being lower after a happy induction. These indicate that mood inductions elicited the desired moods.

The MANOVA also yielded a significant interaction of Induction Type (pre-induction, sad induction, happy induction) × Instruction Type (engaging vs. non-engaging) [Wilks' Lambda = 0.52, *F*_(6, 21)_ = 3.21, *p* < 0.05]. The following univariate ANOVAs indicated that following a sad mood induction, participants who received emotionally engaging instructions exhibited a marginally significant greater decline in general mood [*F*_(1, 26)_ = 3.70, *p* = 0.07] and a greater incline in sad rating [*F*_(1, 26)_ = 6.24, *p* < 0.05] than participants who received emotionally disengaging instructions, compared to baseline mood ratings. No difference was found between groups in happy ratings following the sad films compared to baseline [*F*_(1, 26)_ = 1.29, *ns*]. Following a happy mood induction, participants who received emotionally engaging instructions exhibited a greater incline in happy [*F*_(1, 26)_ = 5.25, *p* < 0.05] and general mood [*F*_(1, 26)_ = 5.34, *p* < 0.05] ratings, and a greater decline in sad rating [*F*_(1, 26)_ = 10.21, *p* < 0.001] than participants who received emotionally disengaging instructions, compared to ratings following the sad induction.

Overall, findings of this study clearly rule out the possibility that high levels of emotional engagement hinder autobiographical retrieval in general, and support the claim that high levels of engagement specifically hinder mood incongruent retrieval. Additionally, results suggest that a happy mood may facilitate recollection of more mood congruent memories than a sad mood, although mood does not influence recall latency of the first mood congruent memory.

## General discussion

In this work, we examined several hypotheses concerning the influence of mood on the fluency of retrieving autobiographical memories opposite to the current mood. According to the “sad valence hypotheses,” fluency of recalling memories opposite to the current mood is expected to be impaired following a sad film but not a happy film (based on Sheppes and Meiran, 2007), whereas according to the “happy valence hypothesis” the fluency of mood incongruent memories is expected to be impaired following a happy film but not as much following a sad film (based on Isen, 1985). According to the “engagement hypothesis,” the degree of emotional engagement impairs fluency of opposite mood memories, thus less fluency of opposite mood memories is expected following high levels of emotional engagement, regardless of mood valence (negative or positive). Our results clearly support the emotional engagement hypothesis and yield some support to the “happy valence hypothesis.” In Study 1, following both the induction of sad and of happy moods, participants who received emotionally engaging instructions had reported their moods to be more strongly affected by the emotional stimuli, had exhibited more than double the recall latency of the first opposite mood memory, and had generated overall less opposite mood memories as participants who received emotionally disengaging instructions. Study 2 yielded similar FAM results inducing only a happy mood, thereby refuting the possibility that results of Study 1 were hampered by order effects. The fact that emotional engagement groups differed in FAM performance but not in self reported mood checks in Study 2 provides evidence that emotional engagement and emotion intensity are separate and independent from one another, and that impairments in mood incongruent retrieval were not due to differences in the intensity of the emotion but rather due to emotional engagement. Results of Study 3 demonstrate that high emotional engagement does not impede autobiographical retrieval in general, but only mood incongruent autobiographical retrieval. Taken together, results indicate that low levels of emotional engagement facilitate mood incongruent retrieval and high levels of emotional engagement hinder mood incongruent retrieval.

Partial support was also found for the “happy valence hypothesis.” In Study 1 fewer mood incongruent memories were generated following a happy film compared to a sad film, and in Study 3, more mood-congruent memories were generated following a happy film compared to a sad film. Mood valence did not affect recall latency of the first memory. The fact that being in a happy mood resulted in fewer mood incongruent memories and more mood congruent memories is rather logical, as individuals in a happy mood are highly motivated to maintain this mood and may be reluctant to recall opposite mood memories (see e.g., Handley et al., 2004).

Findings of the current work regarding emotional engagement support the analogy proposed in the Introduction between mood incongruent retrieval and task switching (Kiesel et al., 2010; Meiran, 2010, see also Greenberg and Meiran, 2013). As in task switching, retrieving memories opposite to the current mood involved a behavioral cost. Moreover, this cost was significantly greater when individuals were more engaged in the emotional experience, as in the phenomenon of “switch asymmetry” (Allport et al., 1994; Yeung and Monsell, 2003). We therefore, suggest that the current findings provide preliminary evidence regarding the link between moods and task-sets.

Various conditions have been found to affect mood incongruent retrieval (see Singer and Salovey, 1988; Rusting, 1998; Holland and Kensinger, 2010 for reviews). We suggest that emotional engagement may be a common mechanism in many of these conditions. Mood incongruent retrieval has been most commonly linked to mood regulation, in which memories opposite in valence to the current unpleasant mood are summoned as attempts to attenuate current mood (e.g., Isen et al., 1978; Isen, 1984; Josephson, 1996). Findings of the current studies demonstrate that such regulation is directly related to emotional engagement since the degree of engagement determines the ability to retrieve mood incongruent memories and therefore to regulate emotions via such retrieval. Likewise, the retrieval of mood incongruent memories is also facilitated when participants are unaware that moods are relevant to the experiment (Parrott and Sabini, 1990). In these scenarios, individuals are likely to disengage from their moods in order to focus on the perceived experimental demands. Lastly, mood incongruent retrieval is facilitated among individuals who tend to avoid negative emotional stimuli (repressors; Boden and Baumeister, 1997), a population which avoids and disengages from negative emotional stimuli by definition. Thus, given the current findings that emotional engagement determines the fluency of mood incongruent autobiographical retrieval, and the above reasoning regarding the plausible involvement of emotional engagement in many of the conditions affecting mood incongruent retrieval, we suggest that degree of emotional engagement provides a parsimonious explanation for the occurrence of mood incongruent memory in many of these conditions.

The current work may bear implications regarding the field of emotion regulation. Although recalling memories opposite in valence to the current mood has been regarded as an effective means of mood regulation (Isen et al., 1978; Isen, 1984; Josephson, 1996), findings of the current work suggest that regulation by means of opposite mood retrieval is difficult and therefore may be inefficient in cases of high emotional engagement. Moreover, individuals suffering from depression may be highly emotionally engaged in feelings of sadness, despair, or guilt (see Clemens, 2007). Following previous observations that individuals suffering from dysphoria and depression often have difficulty in effectively regulating their emotions by various strategies, including retrieval of incongruent memories (Joormann and Siemer, 2004; Joormann et al., 2007), we tentatively suggest that high levels of emotional engagement may be a significant mediating variable in the ability of such individuals to adaptively regulate their emotions by means of mood incongruent retrieval. However, further research is needed in order to better substantiate such a potential mediating role of emotional engagement in depression and other mood disorders.

An additional possible contribution of this study concerns the FAM as a measuring tool in the study of emotion. Results of the current work indicate that although the FAM has originally been introduced as a measure of sadness (Sheppes and Meiran, 2007), it may serve as a more generic measure of emotional engagement, in positive as well as negative emotions. Having such an indirect, behavior-based measure for emotional engagement which is considerably less prone to demand characteristics than many other measures and is independent of self reported emotion which may be biased or inaccurate (Nisbett and Wilson, 1977; Hunt et al., 2003) may prove useful in emotion research.

## Authors' note

This work was partly supported by a research grant to the second author from the Israel Science Foundation.

### Conflict of interest statement

The authors declare that the research was conducted in the absence of any commercial or financial relationships that could be construed as a potential conflict of interest.

## Footnotes

^1^Although this analysis could have been performed in a repeated measures ANOVA, a MANOVA seemed more appropriate, as it may serve as an alternative with a decreased risk of assumption violation (see O'Brien and Kaiser, 1985). Additionally, MANOVA may be more appropriate since, properly speaking, the mood checks were a series of tests rather than the same test measured repeatedly, as assumed in ANOVA.
